# Extending ‘Contact Tracing’ into the Community within a 50-Metre Radius of an Index Tuberculosis Patient Using Xpert MTB/RIF in Urban, Pakistan: Did It Increase Case Detection?

**DOI:** 10.1371/journal.pone.0165813

**Published:** 2016-11-29

**Authors:** Razia Fatima, Ejaz Qadeer, Aashifa Yaqoob, Mahboob ul Haq, Suman S. Majumdar, Hemant D. Shewade, Robert Stevens, Jacob Creswell, Nasir Mahmood, Ajay M. V. Kumar

**Affiliations:** 1 National TB Control Program, Islamabad, Pakistan; 2 Centre for International Health, Burnet Institute, Australia; 3 Department of Community Medicine, Mahatma Gandhi Institute of Medical Sciences (MGIMS), Sewagram, Wardha, India; 4 International Union Against Tuberculosis and Lung Disease (The Union), South-East Asia Regional Office, New Delhi, India; 5 Mott MacDonald, London, United Kingdom; 6 Stop TB Partnership, Geneva, Switzerland; 7 International Union Against Tuberculosis and Lung Disease, Paris, France; Wadsworth Center, UNITED STATES

## Abstract

**Background:**

Currently, only 62% of incident tuberculosis (TB) cases are reported to the national programme in Pakistan. Several innovative interventions are being recommended to detect the remaining ‘missed’ TB cases. One such intervention involved expanding contact investigation to the community using the Xpert MTB/RIF test.

**Methods:**

This was a before and after intervention study involving retrospective record review. Passive case finding and household contact investigation was routinely done in the pre-intervention period July 2011-June 2013. Four districts with a high concentration of slums were selected as intervention areas; Lahore, Rawalpindi, Faisalabad and Islamabad. Here, in the intervention period, July 2013-June 2015, contact investigation beyond household was conducted: all people staying within a radius of 50 metres (using Geographical Information System) from the household of smear positive TB patients were screened for tuberculosis. Those with presumptive TB were investigated using smear microscopy and the Xpert MTB/RIF test was performed on smear negative patients. All the diagnosed TB patients were linked to TB treatment and care.

**Results:**

A total of 783043 contacts were screened for tuberculosis: 23741(3.0%) presumptive TB patients were identified of whom, 4710 (19.8%) all forms and 4084(17.2%) bacteriologically confirmed TB patients were detected. The contribution of Xpert MTB/RIF to bacteriologically confirmed TB patients was 7.6%. The yield among investigated presumptive child TB patients was 5.1%. The overall yield of all forms TB patients among investigated was 22.3% among household and 19.1% in close community. The intervention contributed an increase of case detection of bacteriologically confirmed tuberculosis by 6.8% and all forms TB patients by 7.9%.

**Conclusion:**

Community contact investigation beyond household not only detected additional TB patients but also increased TB case detection. However, further long term assessments and cost-effectiveness studies are required before national scale-up.

## Introduction

Pakistan ranks fifth amongst the 22 high burden tuberculosis (TB) countries with TB incidence rate of 270 per 100,000 population and prevalence of 341 per 100,000 population; however, only 62% of these cases are detected and reported to the National TB Program (NTP) [1]. Eighteen national prevalence surveys in high TB burden countries have demonstrated that more than half of TB patients remain undetected [2]. Progress towards achieving national and global TB targets requires detecting these ‘missed’ cases. To achieve this, the current strategy of passive case finding (PCF) will not be sufficient. There has been renewed interest and investment in for systematic screening / intensified case finding strategies for TB [2].

A systematic review of 62 studies concluded that screening increased the number of TB patients found in the short term and found patients earlier with less severe disease [3]. ACF strategies include mobile screening units, contact investigation, public-private collaborative activities, and mass community screening. Indiscriminate mass screening of communities for TB is not recommended due to concerns of cost and lack of evidence of effectiveness [4–6]. Many ACF initiatives among high risk groups including household and close contacts and people living in urban slums have shown promising results [4,7–10]. However, the evidence of impact on TB transmission and epidemiology (incidence, prevalence and mortality) is insufficient as there is dearth of studies with long term follow up and the availability of a control group [3]. Operational research on well planned ACF interventions should be conducted to measure the impact of ACF and inform NTPs and policy makers.

Recognizing the need to screen high risk groups, the Pakistan NTP adopted several novel strategies [11]. One such innovative strategy was TB screening among close community contacts living within a radius of 50 metres of index TB cases in addition to household contact investigation. All contacts were screened for TB symptoms and investigated using smear microscopy, Xpert MTB/RIF (for smear-negatives) and chest radiography. A similar concept of radius contact screen has previously been tried successfully in patients of smallpox [12]. The NTP implemented this with the support of TB-REACH wave III in four districts of Punjab province, Pakistan, from July 2013 to June 2015.

This operational research is the first systematic assessment of the impact of close community contact screening on TB case detection in Pakistan. **Specific objectives** were i) To assess the number of household and community contacts of sputum smear-positive TB patients screened in intervention districts between July 2013—June 2015; and among them, to determine the number (proportion) of presumptive TB patients identified, TB patients detected and number initiated on treatment and ii) to determine the percentage increase in TB case detection relative to baseline (July 2011 –June 2013) in the intervention districts.

## Methods

### Ethics Approval

The study protocol was reviewed and approved by the Ethics Advisory Group of International Union Against Tuberculosis and Lung Disease (The Union), Paris, France. Sharing of the patient-wise data due to concerns of confidentiality and other ethical restrictions is imposed by the advisory group (eag@theunion). Permission was taken from NTP, Pakistan to conduct this operational research. As this research involved analysis of secondary data collected routinely under the national programme, the need for individual informed consent was waived by the ethics committee.

### Study design and population

This research involved a retrospective record review of a cohort of community contacts living within 50 metre radius of infective tuberculosis patients in intervention districts. For the first objective, study participants were all the community contacts within 50 metre radius of a sputum smear-positive TB patient registered for treatment in intervention districts from July 2013 to June 2015. For the second objective, study participants were all notified TB patients in intervention districts during pre-intervention (July 2011-June 2013) and intervention period (July 2013-June 2015).

### Setting

#### General

Thirty-six percent of Pakistan’s 182.5 million people live in urban areas. Pakistan is a country with high and growing proportion of people living in urban slums[13]. People living in these overcrowded settings are more likely to have low nutritional status and are vulnerable to communicable diseases, in particular TB. The Basic Management Unit (BMU) is a health facility providing primary health care, TB diagnosis with sputum smear microscopy, patient registration and treatment. The Xpert MTB/RIF test is available at Programmatic Management Drug Resistant Tuberculosis (PMDT) sites for Multi Drug Resistance MDR contacts and treatment failure (all category II patients). Household contact investigation of smear positive index TB patients is routinely implemented by the NTP.

#### Study setting

Four districts with a high concentration of slums were selected as intervention areas which included Lahore, Rawalpindi, Faisalabad and Islamabad. These districts have a total population of 18 million half of whom live in slums.

#### Intervention

**Fig 1:**
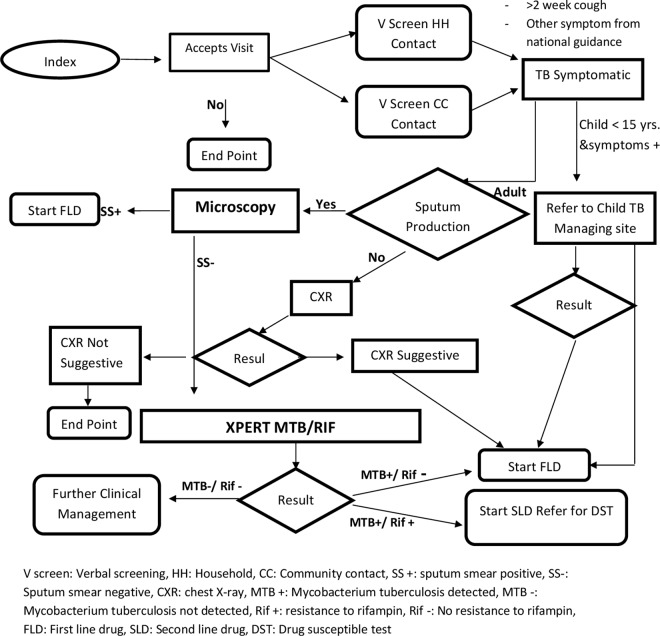
Flow chart to explain the flow of patients in TB Reach project, Pakistan

All people staying within a radius of 50 metres from the household of index patient were contacted and screened for TB. A cut-off of 50 metres was chosen based on the data from the electronic TB surveillance system which revealed the presence of many patients coming from the same family, same address or neighbouring areas and suggested high rates of epidemiological (geographical) clustering of TB patients. Additionally, the approximate number of household in this radius (5–50) was deemed feasible to be covered under close community screening by the NTP. Mobile phones enabled with Geographic information system (GIS) technology were used by field workers to identify households within a 50 metre radius of the index patient and collect data. All people permanently residing within a radius of 50 metres, available at the time were contacted and screened for TB. Measures were taken to maintain the index patient’s confidentiality in front of contact persons. Any person with productive cough for more than two weeks was defined as ‘presumptive TB patient’. Initially, one spot sputum sample was collected and transported to the BMU for diagnostic testing. If it was found negative, another visit was made to collect second sample for Xpert MTB/RIF. The project diagnostic algorithm is displayed in **Fig 1**. According to project algorithm, smear-positivity was defined as at least one smear containing at least one acid-fast bacillus, which is in tune with WHO guidelines. All patients with a positive smear, or M. tuberculosis detected by Xpert were considered bacteriologically positive TB. Clinical TB was defined as active TB diagnosed by a physician who decided to give a full course of TB treatment based on clinical, radiology and/or histology findings but without bacteriological confirmation.

Patients that were bacteriologically positive for TB were contacted by the project Field Officer (FO) and referred to the nearest BMU for registration and treatment initiation. All presumptive child TB patients aged less than 15 years were referred to hospitals with availability of specialist paediatric care for diagnosis and treatment and were followed-up. People who were negative on both sputum microscopy and Xpert MTB/RIF were referred to the nearest BMU for follow-up according to national guidelines.

Xpert MTB/RIF testing was funded by the project and provided free of cost. Sixty Field Officers (FOs) were trained under this project and allocated to BMUs. Primary and maternal health workers called Lady Health Workers (LHW) from respective BMUs accompanied the FOs during the house to house visits. The activity of LHW was incentivized by the project. FOs were provided two wheeler transport facility to transport the sputum to the BMU. FOs were equipped with mobile phones enabled with GIS and web interface technology for data collection. Web-based system was developed to capture data on mobile phones which were uploaded real-time to a centralized server. This was enabled by GIS technology which captured the co-ordinates of the location of the households of the index case as well as the household and community contacts. The web-based interface permitted comprehensive daily real-time supervision of field officers’ performance, with no data loss. The online database was password protected and only authorized persons were permitted to access the data for monitoring and analysis purposes.

### Data collection and variables

For the first objective, aggregate district-wise (quarter-wise) data was collected: number of persons identified for screening; stratified by household and community; age and sex distribution; presumptive TB patients (yes/no); sputum collection (yes/no); result of sputum microscopy (positive, negative and pending); outcome of Xpert MTB/RIF test (MTB detected/ rifampicin resistance/ MTB not detected/ No result or error or invalid); TB status (yes/no); and treatment initiation (yes/no). For the second objective the following aggregate data was collected: total number of TB patients notified during pre-intervention and intervention period, stratified by district, type of TB, age distribution and sex distribution.

### Data Analysis

Data was extracted out of the centralized database and analysed in STATA v12 (StataCorp, TX 2011). Descriptive aggregate data analysis was done to assess the number of people screened in household and community and among them, to determine the number (proportion) with presumptive TB and diagnosed as TB. Number needed to screen (NNS—overall and stratified by household contacts and close community contacts) to detect one TB patient was calculated among total contacts screened and among presumptive TB patients. The percentage increase in case notification in intervention districts was calculated between pre-intervention (Jul 2011-Jun 2013) and intervention periods (July 2013-June 2015).

## Results

A total of 783043 contacts were screened for tuberculosis symptoms, of whom 89222 were household contacts and 693821 were close community contacts. A total of 23741 (3.0%) presumptive TB patients were identified and of whom, 14973 (63.1%) presumptive TB patients were investigated for sputum microscopy and 5019 (21.1%) underwent chest X-ray. Among investigated, 4084 (20.4%) were bacteriologically confirmed TB patients. Of 11064 found to be smear-negative and requiring an Xpert test, only 6877 (62.2%) were tested using Xpert MTB/RIF. Of them, 522 (7.6%) were found to have TB and among them, 44 (8.4%) had rifampicin resistance **(Table 1).** A total of 5019 presumptive TB patients were examined through chest X-rays and among them 559 (11.1%) were clinically diagnosed as TB patient based on chest X-ray. Of the 4710 TB patients detected as a result of this intervention, 4604 (97.7%) were initiated on treatment.

**Table 1:** Screening Yield and Number Needed to Screen among household contacts and Close community contacts of index patients in intervention* districts**, Pakistan.

Contacts screening	Index household	Close community within 50m	Total
		radius of index HH	
**Total screened**	89222	693821	783043
Presumptive TB patients	5173 (5.8%)	18568 (2.7%)	23741 (3.0%)
Investigated for sputum smear	3356/5173 (64.9%)	11617/18568 (62.6%)	14973/23741 (63.1%)
microscopy			
Number smear positive	932 /3356 (27.8%)	2977/ 11617 (25.6%)	3909/14973 (26.1%)
Number smear negative eligible	2424/3356 (72.2%)	8640/ 11617 (74.4%)	11064/14973 (73.9%)
for Xpert testing			
Number tested using Xpert	1476/2424 (60.9%)	5401/8640 (62.5%)	6877/11064 (62.2%)
Number of TB positive on Xpert	160/1476 (10.8%)	362/5401 (6.7%)	522/6877 (7.6%)
Number of Rifampicin Resistant	14/160 (8.8%)	30/362 (8.3%)	44/522 (8.4%)
positive			
Number who underwent Chest	992/5173 (19.2%)	4027/18568 (21.7%)	5019/23741 (21.1%)
X-ray			
Number clinically diagnosed as	133/992 (13.4%)	426/4027 (10.6%)	559/5019 (11.1%)
TB based on Chest X-ray			
**Bacteriologically confirmed**	996/5173 (19.3%)	3088/18568 (16.6%)	4084/23741 (17.2%)
**TB**			
NNS among total screened	90	225	192
NNS among presumptive TB	5	6	5.8
**All forms of TB**	1154/5173 (22.3%)	3556/18568 (19.1%)	4710/23741 (19.8%)
• Lahore	479 (41.5)	1540 (43.3)	2019 (42.9)
• Faisalabad	363 (31.5)	1279 (36.0)	1642 (34.9)
• Rawalpindi	277 (24.0)	668 (18.8)	945 (20.0)
• Islamabad	35 (3.0)	69 (1.9)	104 (2.2)
NNS among total screened	77	195	166
NNS among presumptive TB	5	5	5.0
Treatment initiated	1126 (97.6%)	3478 (97.8%)	4604 (97.7%)
**Child TB patients among child**	84/1269 (6.6%)	213/4596 (4.6%)	297/5865 (5.1%)
**presumptive**			
NNS among total child screened	358	1143	921
NNS among presumptive child	15	22	20
TB patients			

*Community screening in addition to household screening within 50 meter radius of index house

** Lahore, Faisalabad, Rawalpindi and Islamabad

NNS to detect one bacteriologically confirmed TB among total contacts screened was 192 overall: 90 among household contacts and 225 among close community contacts. NNS to detect one confirmed TB (all forms) among total contacts screened was 166 overall: 77 among household contacts and 195 among close community contacts. The screening yield for all forms TB patients among investigated presumptive TB patients was 19.8% overall: 22.3% among household contacts and 19.1% among close community contacts. However, there was inter-district variation in case detection, which was highest in Lahore (42.9%) and Faisalabad (34.9%).

NNS to detect one child TB patient among total child contacts (<15 years) screened were 921 overall: 358 among household contacts and 1143 among close community contacts. The screening yield for child TB patients among investigated presumptive child TB patients was 5.1%.

**Table 2**shows age, sex and bacteriologically positive status of TB patients notified during the intervention period and compared with the pre-intervention period. Overall, there was an eight percent increase in notified TB patients during the intervention period. This was consistent among males or females, children or adults and sputum positive TB. However, there was inter-district variation and the increase in case detection was highest in Lahore district (9.1%).

**Table 2:** Numbers of tuberculosis patients reported in the pre-intervention and intervention period of the project (Close community contact screening) in Pakistan.

	Pre-intervention	Intervention	Increase in TB case
	(July 11–June 13)	(July 13 –June 15)	detection
	n	n	%
**Lahore**	49778	54325	9.1
**Faisalabad**	20576	22220	8.0
**Rawalpindi**	27181	28881	6.3
**Islamabad**	2849	2915	2.3
**0–4 yrs**	1487	1591	7.0
**5–14 yrs**	11019	11697	6.2
**> = 15 yrs**	87878	95143	8.3
**Male**	51013	55178	8.2
**Female**	49371	53263	7.9
**Bacteriological positive**	28159	30066	6.8
**Total all forms**	**100384**	**108341**	**7.9**

**Fig 2:**
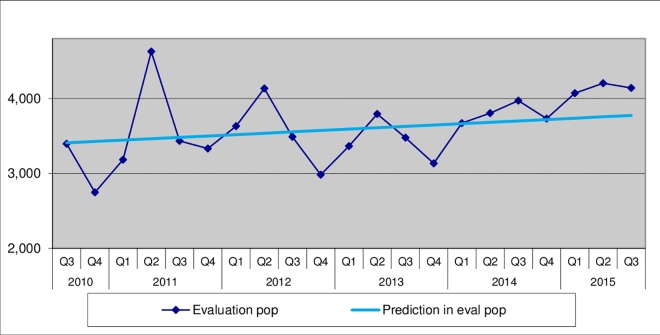
New sputum positive TB case notifications (NSP) compared with projected baseline linear trends

Fig 2 demonstrates that there was an increase in new sputum positive (NSP) case notifications during the project above than the expected linear trend in the intervention districts.

## Discussion

Community contact investigation within 50 metres of an index patient as an ACF strategy which included household contact investigation and contact investigation beyond household resulted in detection of additional TB patients and increased case notification during intervention period by about 7.9% in Pakistan. This research had several strengths. First, community contact investigation within 50 metres is a novel ACF intervention that to our knowledge has not been reported in literature. Second, the intervention was conducted on a large population in urban Pakistan, hence generalizable to similar settings and TB epidemics. Extending the contact circle seems obvious in overcrowded areas, and fairly easy to perform. Third, this was the first time the Xpert MTB/RIF test was implemented in contact screening in Pakistan. Fourth, the GIS web-based interface was used for data collection which permitted comprehensive daily real-time monitoring and supervision and ensured accuracy and completeness of data. Fifth, despite being implemented by project staff, the intervention was conducted routinely under the stewardship of Pakistan NTP and thus feasible, if considered for scale up. Finally, the study adhered to the STROBE guidelines for reporting [14].

The study had several limitations. No data was collected on participants who did not consent or were not available at the time of field visit in intervention areas; and it was possible that this participant sample was not representative and had different characteristics. However, it was not operationally feasible to make a second visit. Details of time to diagnosis from onset of symptoms and visit to a health care facility for TB symptoms (if any) were not collected in this study. Treatment outcomes were not compared among those detected through the intervention. This is being studied and will be reported in a separate paper. However, a recent systematic review has found no difference in treatment outcomes between TB patients found by screening and those found through PCF [3]. Evidence from clinical trials is lacking and more data are needed to inform international policy. Most of TB diagnoses were based on a single smear with no culture that could lead to false positive TB cases which is a common concern with regards to ACF [15].

Xpert MTB/RIF offers a new possibility for diagnosis of bacteriological positive TB among those initially negative on sputum examination [16]. TB yield from Xpert MTB/RIF was lower in intervention, but more absolute numbers of MDR TB diagnosed; which has benefit in stopping transmission if linked to treatment and care.

Contact tracing is a proven strategy to identify infected individuals, and a vital component of any tuberculosis (TB) elimination strategy [17,18]. Community based screening programmes in high burden TB countries have mainly relied on symptom screening, sputum smears and culture, due to the logistical and operational challenges of mass CXR screening [5,19]. Various ACF strategies among high risk groups have shown positive results in identification of TB [7–9]. Nonetheless, the evidence that ACF impacts on TB epidemiology remains weak. The ZAMSTAR evaluated two different interventions (respectively TB household visits and community-wide case finding) and reported a significant reduction in undiagnosed TB at community level from the household intervention [19]. A study from Cambodia provided evidence of reduced TB notifications among individuals who underwent intensive screening for TB over 2 years of follow-up [20]. However, a study from Zimbabwe showed increased case notification rates during the study period, with a 41% reduction in TB prevalence in 3 years of implementation of community-based TB case finding [5]. A systematic review reported that screening contacts does not contribute to more than 9% of all notified patients [3]. In current study, of all the bacteriologically confirmed TB patients diagnosed in the intervention period, 75% were contributed by close community screening. NNS to find one patient of TB was 90 in household contacts and 225 in community contacts, where a systematic review reported the NNS was reported 100 in community contacts in high burden TB countries [21].

The high screening yield among presumptive TB patients in beyond household contacts (19.1%) and in household contacts (22.3%) justified the intervention. Similarly in another study in Sindh, Active case detection through household contact investigation have found 22.7% yield of TB patients [22]. Other studies on active case detection have found yields of TB patients among those referred ranging from 4% in South Africa [23], 13% in Ethiopia [24] and 15.5% in Pakistan [7]. 297 additional children with TB were detected due to intervention and this generated the evidence that the project contributed to the overall increase in child case detection but the NNS was greater than adults, this suggested that the project may have missed child patients that should be addressed while scaling-up this intervention. Increase in patients notified was only 2.3% in Islamabad district which needs to be further explored.

### Policy implication

The close community investigation is feasible to conduct though the main operational implications are the need for additional human resources and access to Xpert MTB/RIF for TB diagnosis. Once the long term impact and cost effectiveness of close community investigation is established, and the feasibility of implementation in to routine activities is considered [25], national programme managers may consider scaling up this intervention nationwide. As of now, the current research provides robust evidence for short term impact of close community investigation on TB case detection.

In conclusion, an innovative strategy involving close community contact tracing using Xpert MTB/RIF led to increase in TB case detection suggesting the value of expanded contact investigation into the community beyond the household. Further studies are needed to establish feasibility and cost-effectiveness before scale-up and assess long-term epidemiological impact.
